# Hygroscopic Mg–Al LDHs composite microspheres for highly efficient hyperspectral camouflage in the VIS and NIR wavebands

**DOI:** 10.1038/s41598-024-66538-4

**Published:** 2024-07-08

**Authors:** Zixun Xie, Le Yuan, Xiaolong Qing, Yaqing Wang, Xiaoyan Wu, Xiaolong Weng

**Affiliations:** 1 Key Laboratory of Materials and Surface Technology (Ministry of Education), School of Materials Science and Engineering, Xihua University, Chengdu, People’s Republic of China; 2 State Key Laboratory of Electronic Thin Films and Integrated Devices, University of Electronic Science and Technology of China, Chengdu, People’s Republic of China; 3 Institute of Fluid Physics, China Academy of Engineering Physics, Mianyang, People’s Republic of China

**Keywords:** Hyperspectral camouflage, Layered double hydroxide, Composite microspheres, Visible and near-infrared spectrum, Moisture absorption, Chemistry, Materials science, Optics and photonics

## Abstract

In order to enhance the hyperspectral camouflage efficacy of stealth coatings against a natural vegetative backdrop, LiCl, known for its significant hygroscopic properties, was incorporated into green Mg–Al layered double hydroxide (Mg–Al LDHs) material. Micron-sized composite microspheres were subsequently synthesized via the spray-drying granulation technique. The structure, morphology, and chemical composition of these microspheres were thoroughly characterized by X-ray diffraction, scanning electron microscopy, laser particle size analysis, nitrogen adsorption–desorption isotherms, and Fourier-transform infrared spectroscopy. The effect of LiCl content on the moisture absorption capacity and near-infrared reflectance spectra of the microspheres was systematically evaluated. We found that incorporating an optimal amount of LiCl into the internal pores of the Mg–Al LDHs microspheres did not compromise their smooth surface morphology and uniform particulate distribution. Notably, when the LiCl content was 10%, the maximum saturation moisture uptake ratio of the coating increased to 0.75 g/g. This hygroscopicity significantly enhanced the absorption and scattering of near-infrared radiation by the coating while concurrently improving its ability to modulate the shape and reflectance of both the visible and near-infrared spectral curves. Spectral congruence between the synthetic coating and natural green foliage was quantified at 97.41%. Moreover, this performance was maintained over 10 cycles of programmed drying and re-humidification, and the coating consistently demonstrated stable hygroscopic properties and sustained over 95% spectral congruence. These optimized artificial coatings were found to effectively confuse hyperspectral classification algorithms, thus blending seamlessly into a natural foliage backdrop. This study provides a new method for regulating VIS and NIR spectral (visible–near infrared spectrum) features, which will be critical for applications in advanced hyperspectral camouflage materials.

## Introduction

The unique visible and near-infrared spectral characteristics of natural leaves rely on their leaf structure and biochemical composition. Most green leaves have inherently similar coloration and spectral characteristics, resulting in four typical reflectance spectrum characteristics in the visible and near-infrared wavebands^1^. The peak centered around 550 nm typically originates from the attenuation of reflected light due to in vivo chlorophyll and carotenoid absorption, and this reflectance signature gives leaves their dominant green hue. Another feature is the abrupt change in reflectance between 680 and 780 nm, known as the red edge, with red edge formation resulting from narrow Q-band absorption of in vivo chlorophyll and internal reflection at the cell walls^2^. The near-infrared plateau is typically found in the 780–1300 nm waveband, characterized by high reflectivity due to the strong near-infrared reflection of porous parenchyma tissue (spongy tissue). Additionally, two distinct water absorption valleys form at 1450 nm and 1940 nm due to the strong near-infrared absorption of cell fluid in the leaves^3^. Moreover, the spectral reflectance of the entire near-infrared waveband will gradually decrease with increasing wavelength.

The high spectral similarity in green leaves is a critical requirement for modern camouflage materials, especially for countering hyperspectral target detection, as they possess the capability to capture subtle differences in solar reflectance spectra between targets and background vegetation. In the natural world, stick and leaf insects have mastered the spectral simulation of foliage. Their coloration and spectra can be adapted to match the growth stages, seasons, and species of leaves in their habitats, thereby perfecting their camouflage. By contrast, simulating the spectral characteristics of green leaves using artificial materials such as camouflage coatings remains highly challenging, especially considering the recent extension of the hyperspectral detection range to the near-infrared waveband (400–2500 nm). Currently, most studies on artificial spectral simulation of green leaves have primarily focused on using extracted chlorophyll and commercial colorants (dyes or pigments), with a focus on accurately replicating the color and visible spectral characteristics. The artificial material can generate human-like visual appearance under specified lighting conditions, thus meeting camouflage requirements in terms of chroma value. Although high visible spectral similarity and controllable color difference have been achieved, significant differences remain in structure and composition between artificial materials and green leaves, which significantly differ from those of natural leaves in the near-infrared spectra. Therefore, this has become a new feature for detection and recognition.

The principal challenge in the advancement of hyperspectral camouflage materials resides in the precise replication of the water absorption valley of leaf cell sap in the 780–2500 nm waveband while concurrently maintaining other spectral characteristics. Liu et al. employed water-based polyurethane as the base material to fabricate a hyperspectral biomimetic material with spectral similarity surpassing 96.9%^4^. However, this material demonstrated susceptibility to degradation due to expansion upon water absorption. Yang et al. encapsulated chlorophyll and water in a multilayered polyethylene film material, effectively replicating the spectral curve of leaves in the visible and near-infrared wavebands. However, this film was susceptible to fracturing, and the chlorophyll was prone to rapid degradation, which led to an inability to maintain spectral characteristics over time^5^. Lv et al. integrated high moisture-absorbing mesoporous molecular sieves into camouflage coatings to emulate the water absorption valley feature of leaves. However, the near-infrared spectral reflectance of the material was markedly higher than that of actual leaves^6^.

Hydrotalcite(LDHs) material consists of a layered double hydroxide compound comprising interlayer cationic laminae and interlayer anions. Because LDHs laminae are rich in hydroxyl groups, and the interlayer can store water molecules, LDHs exhibit an evident water absorption valley feature in the near-infrared band^7^, with this characteristic analogous to the near-infrared spectrum of leaves. In recent years, with the interpenetration of various disciplinary fields, LDHs have been widely used in electrodes^8^, adsorbents^9^, UV-blocking materials^10^, infrared-absorbing materials^11^, catalysis^12^, and wastewater treatment^13^. However, the research used in the field of hyperspectral camouflage is quite scarce. For example, Miao et al. prepared a Mg–Al LDHs coating to be used in the field of hyperspectral camouflage, but the maximum saturation moisture uptake ratio of such a coating in the natural environment is only 0.115 g/g, and the reflectivity intensity of the water absorption valley can not be further reduced, so that it is not possible to fine-tune the simulation of the characteristics of water absorption valley in the spectra of the plants in the humid region^14^. Yuan et al. incorporated various colored anions into the interlayer of Mg–Al LDHs, successfully emulating the visible and near-infrared spectral shape of leaves by adjusting the type and ratio of interlayer anions. The resemblance of the spectral profile to that of leaves reached 94%, with the material exhibiting a wide color adjustment range and high temperature resistance. Nevertheless, the low hydroxyl/water content of the material led to insufficient absorption in the near-infrared spectrum, resulting in reflectivity that was significantly greater than that of leaves, which constrained its utility for hyperspectral camouflage applications.

LiCl serves as a hygroscopic agent with a potent water-absorbing ability^15^ and is frequently employed to augment the hygroscopic capacity of various organic/inorganic composites. The interaction mechanism of LiCl with water vapor encompasses solid/gas hydration reactions, gas/liquid/solid deliquescence processes, and liquid/gas absorption phenomena^16^. Sun et al. incorporated 30 wt% LiCl into the porous matrix of UiO-66 via a wet impregnation technique, which could increase the water absorption of the composite by up to eight times^17^. Hou et al. synthesized a porous graphene aerogel fiber imbued with a LiCl hygroscopic agent through wet spinning, which significantly enhanced the material's microwave absorption capacity and bandwidth by leveraging the water-absorbing properties of LiCl^18^. To elevate the near-infrared spectral congruence between a camouflage coating and leaves, this study infused a LiCl hygroscopic agent into a green Mg–Al LDHs material to tailor composite microspheres with a micron-scale particle size using the spray granulation technique. The effects of LiCl content on the structural integrity, morphological features, water content, and spectral characteristics of the composite microspheres were rigorously investigated. The influence of the composite microspheres on the spectral reflectance and hyperspectral camouflage efficacy of the camouflage coating was subsequently confirmed. It is promising to achieve a refined simulation of plant spectra by adjusting the ratios of LiCl to Mg–Al LDHs.

## Experimental

### Materials

Magnesium chloride, aluminum chloride hexahydrate, ethylene glycol, sodium hydroxide, oleic acid, anhydrous lithium chloride, ethyl acetate, butyl acetate, xylene, silane coupling agent KH560 were purchased from Chengdu Cologne Chemical Co., LTD. Alizarin green, acid yellow dyes were purchased from Tianjin Duofuyuan Industrial Co., LTD. Hydroxy acrylic resin produced by Elementis Deuchem (Shanghai) Chemical Co., Ltd.

### Sample preparation

#### Preparation of LiCl-LDHs composite microspheres

The Mg–Al layered double hydroxides (LDHs) were synthesized using a classical co-precipitation method. The synthesis commenced by dissolving 0.3 mol of MgCl_2_ and 0.1 mol of AlCl_3_·6H_2_O in 500 ml of deionized water, with continuous magnetic stirring until complete dissolution. Subsequently, a specific volume of dye solution, composed of alizarin green, acidic yellow, and deionized water in a mass ratio of 1:21:40, was added. Following this, a 0.8 M sodium hydroxide solution was incrementally added to the mixture to adjust the pH of the reaction solution to 10. After the reaction concluded, the mixture could age for an additional 4 h, followed by washing with deionized water and drying to yield green Mg–Al LDHs powder.

In the preparation of LiCl-LDHs composite microspheres, 500 g of Mg–Al LDHs powder was dispersed in 4.5 L of deionized water, and subsequently milled to form a slurry with a 10 wt% concentration at a rotational speed of 2000 rpm using a sand mill. During the milling process, an additional x wt% of anhydrous lithium chloride was introduced. Ultimately, composite microspheres with an x% LiCl-LDHs content were produced through a centrifugal spray granulation technique, where the atomization frequency was carefully adjusted to 25 Hz.

#### Preparation of hyperspectral camouflage coatings

A hyperspectral camouflage coating was developed, incorporating x% of LiCl-LDHs composite microspheres as the functional fillers and using acrylic resin as the binder. Initially, the acrylic resin was mixed with a predetermined amount of diluent, followed by the addition of 0.5 wt% KH560 silane coupling agent and oleic acid. Subsequently, 360 wt% of the microsphere powders and 0.1 wt% of a curing agent were incorporated into the mixture. This blend was then thoroughly dispersed with a homogenizer. Finally, the formulation was applied in an even layer onto the aluminum sheet via an air spraying method and allowed to cure at room temperature for 24 h.

### Characterization and measurements

The sample composition and phase structure were characterized using a Bruker D2 X-ray diffractometer, which utilized monochromatic Cu Kα radiation (λ = 0.15406 nm) at settings of 40 kV and 30 mA, featuring a step size of 0.02° and a scanning speed of 5° per minute. The particle size distribution was investigated using an LS-POP(9) laser particle size analyzer. The sample particle morphology was observed with a FEI QUANTA 250 field emission scanning electron microscope. Adsorption/desorption BET curves were evaluated using a Micromeritics ASAP2460 surface area and porosimetry analyzer. Infrared transmission spectra spanning 4000–400 cm^−1^ were recorded using a BRUKER Tensor 27 Fourier-transform infrared spectrometer. The reflectance spectra for the powder samples, within the wavelength range of 350–2500 nm, were assessed with a PerkinElmer Lambda 750 UV–VIS–NIR spectrophotometer.

The determination of the water content in composite microsphere powder and coating materials proceeds as described below. Initially, samples are subjected to drying in an oven at 100 °C until they reach constant weight, indicative of the complete removal of moisture and impurities. This weight is designated as *m*_1_. Subsequently, samples are transferred to a controlled environment chamber set at 20 °C and 99% humidity, where they are left until water absorption equilibrates, at which point the weight, denoted as *m*_2_, is measured. Utilizing the mass measurements before and after moisture exposure, the saturated adsorptive capacity (*Q*, expressed in g/g), is calculated using formula (1).

1 $$Q{ = }\frac{{\left( {m_{2} - m_{1} } \right)}}{{m_{1} }}$$

To evaluate the spectral congruity between the sample and the plant leaves, the spectral correlation coefficient, as defined by Eq. (2), was utilized.

2 $$r_{xy} = \frac{{\sum\nolimits_{i = 1}^{m} {\left( {x_{i} - \overline{{x_{i} }} } \right)\left( {y_{i} - \overline{{y_{i} }} } \right)} }}{{\sum\nolimits_{i = 1}^{m} {\left( {x_{i} - \overline{{x_{i} }} } \right)^{2} \sum\nolimits_{i = 1}^{m} {\left( {y_{i} - \overline{{y_{i} }} } \right)^{2} } } }}$$

In this context, *x*_*i*_ represents the spectral value attributed to the sample, while *y*_*i*_ corresponds to the spectral value of the plant leaves. $\overline{{x_{i} }}$ and $\overline{{y_{i} }}$ denote the respective average spectral values. If the material's spectrum is identical to that of the plant leaves, the correlation coefficient will be 1. Otherwise, the correlation coefficient will register as less than 1^19^.

3 $$\left\{ {\begin{array}{*{20}l} {\overline{{x_{i} }} = \frac{1}{m}\sum\limits_{i = 1}^{m} {x_{i} } } \hfill \\ {\overline{{y_{i} }} = \frac{1}{m}\sum\limits_{i = 1}^{m} {y_{i} } } \hfill \\ \end{array} } \right.$$

## Results and discussion

**Figure 1:**
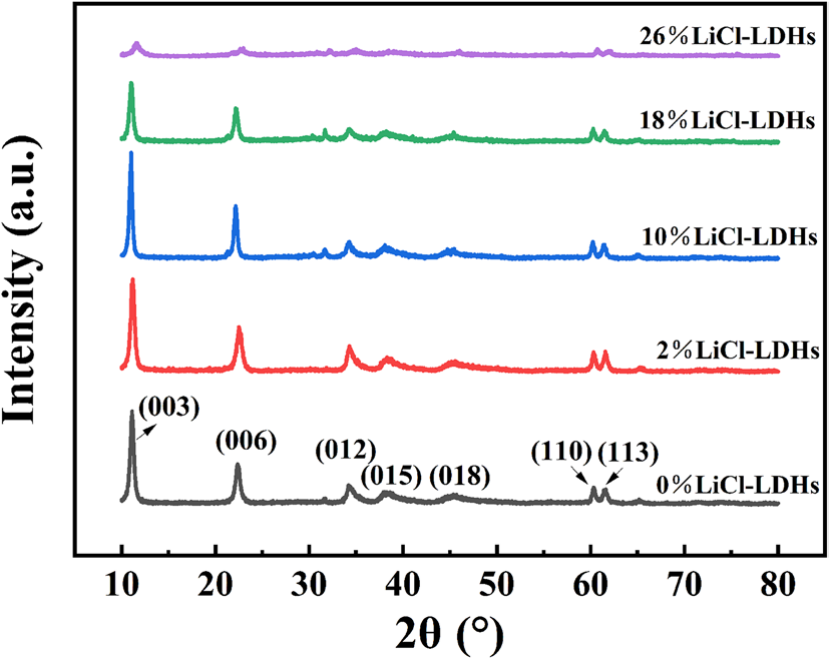
XRD pattern of LiCl-LHDs composite microspheres with different LiCl content.

Figure 1 presents the XRD analysis of the LiCl-LDHs composite microspheres, revealing the characteristic diffraction peaks of the Mg–Al LDHs in the sample’s XRD pattern^20^. Specifically, the peaks near 11° and 23° aligned with the (003) and (006) crystal planes of the Mg–Al LDHs, demonstrating the quintessential peaks of hydrotalcite. Additionally, the absence of diffraction peaks from extraneous substances suggested that LiCl was mechanically intermixed with Mg–Al LDHs in an amorphous state, rather than reacting with them^21^. The incorporation of a suitable quantity of LiCl was found to enhance the crystallinity of the LDHs, achieving optimal crystallinity at a LiCl composition of 10%. However, an increase in LiCl content beyond this point led to a diminished intensity of the Mg–Al LDHs diffraction peaks. The superfluous amorphous LiCl diminished the relative concentration of the Mg–Al LDHs in the composite. Then, the smaller-sized Li^+^ ions possibly infiltrated the lamellar structure of hydrotalcite, thus disturbing the crystal lattice^22^.

**Figure 2:**
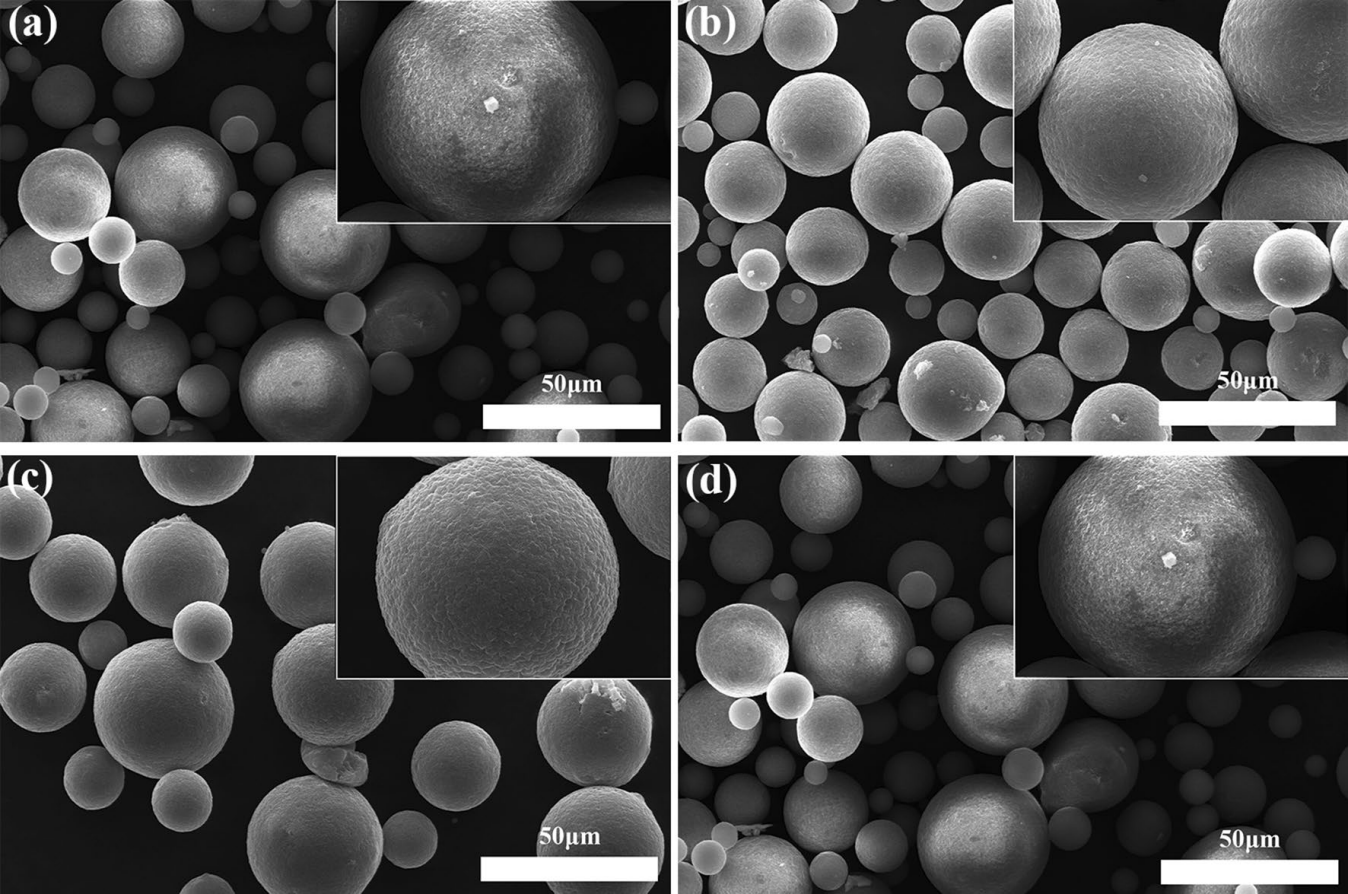
SEM morphology of LiCl-LDHs composite microspheres with different LiCl content [(**a)** 2% LiCl-LDHs; (**b**) 10% LiCl-LDHs; (**c**) 18% LiCl-LDHs; (**d**) 26% LiCl-LDHs].

Figure 2 presents the SEM images of the LiCl-LDHs composite microspheres with different LiCl concentrations, demonstrating that post-spray drying, samples with minimal LiCl content possessed spherical particles characterized by a uniform morphology and smooth exteriors. The particle size exhibited a broad distribution, with the dominance of smaller particles. As the LiCl concentration increased, a marked increase was observed in the larger particles, which was attributed to the spray granulation process transitioning from atomized droplets to solid particles. The water evaporation rate from the droplets dictated the ultimate size and shape of the resultant particles. Given its hygroscopic nature as a salt filler, LiCl extended the evaporation duration of the water within the droplets during spray drying, resulting in enlarged particle dimensions. At excessive LiCl ratios (26%), incomplete drying of the particle exteriors could occur, resulting in interparticle adhesion. Moreover, superabundant LiCl modified the surface tension of the aqueous solution, diminishing the capillary forces that acted upon the particle exteriors and compromising their sphericity.

Moreover, higher LiCl concentrations precipitated on the particle surfaces, influencing the morphological integrity of the composite particles during the drying phase. As shown in Fig. 2, the LiCl-LDHs composite microspheres containing a minimal LiCl fraction (2%) exhibited smooth exteriors with an absence of discernible LiCl precipitates, which was indicative of the intraspherical distribution of lithium salts^23^. Increasing LiCl ratios resulted in an incremental roughening of the composite microsphere surfaces, which was particularly noticeable at an 18% LiCl-LDHs concentration. At a critical threshold of 26% LiCl, the microsphere surfaces were noticeably enriched with LiCl particulates. Concurrently, the composite particles displayed an uneven morphology, with a significantly increased tendency for agglomeration^24^.

**Figure 3:**
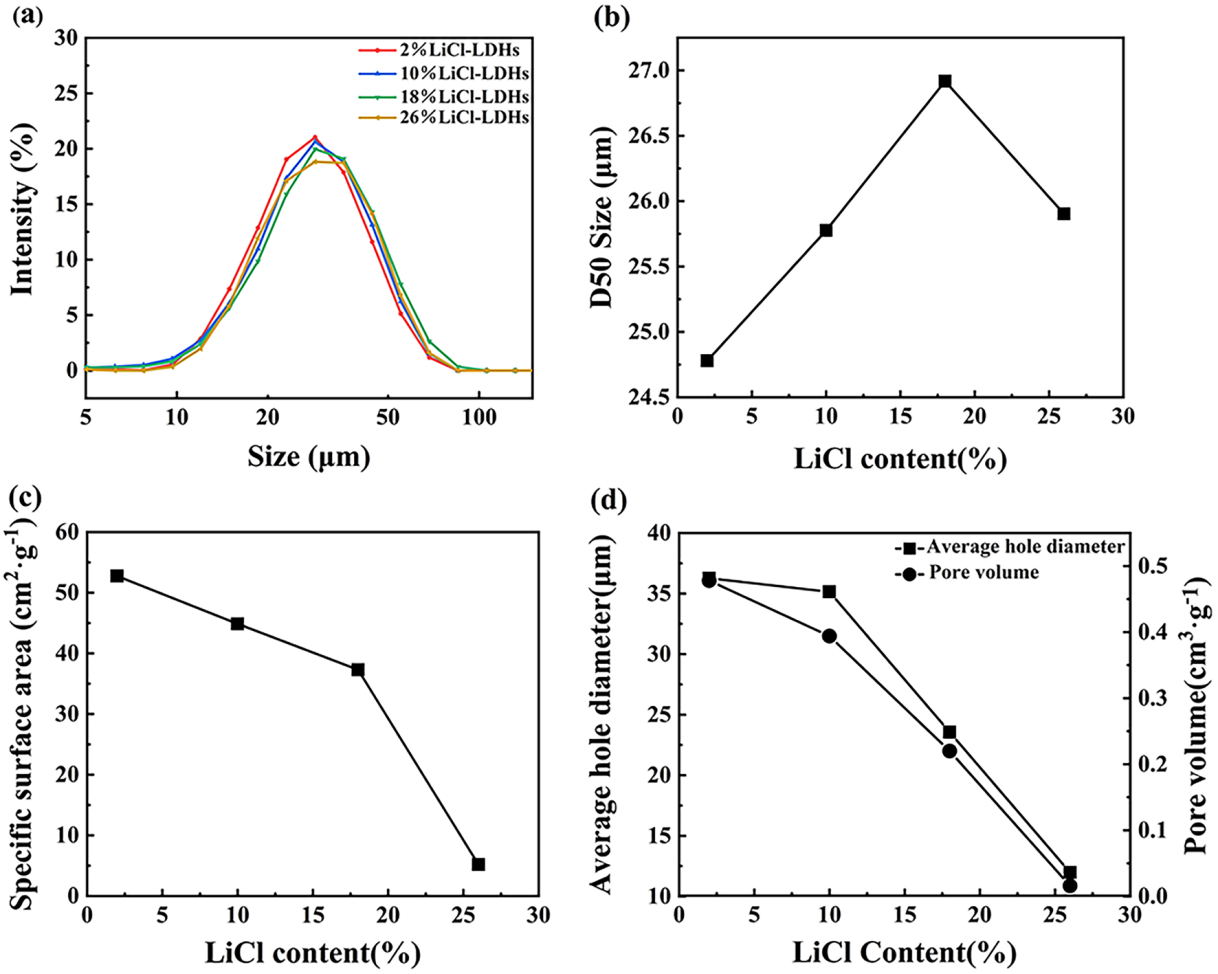
Particle size distribution (**a**), D50 particle size (**b**), specific surface area (**c**) and average pore size (**d**) of LiCl-LDHS composite microspheres with different LiCl content.

Figure 3 presents the particle size distribution and specific surface area measurements of the LiCl-LDHs composite microspheres. Particle sizes resulting from spray granulation ranged from 10 to 90 μm, demonstrating a wide distribution. The particle size distributions of the samples showed minor variations in response to different LiCl concentrations. Analysis of the median particle size (D_50_) data, as illustrated in Fig. 3b, suggested that LiCl incorporation decreased the water evaporation rate in spray drying, resulting in a slight increase in microsphere size with higher LiCl levels. At an 18% LiCl concentration, the median particle size (D_50_) of the sample increased to 26.9 μm. However, further increases in LiCl content led to a significant reduction in particle size, which could be attributed to the compromised spherical morphology.

The internal architecture of the composite microspheres was also examined using specific surface area analyses. Figure 3c demonstrates a significant decrease in the specific surface area of the composite microspheres as the LiCl content increased, particularly at higher concentrations. When correlated with the SEM images and particle size distribution data, it was clear that variances in external size and morphology were minor across the range of 2–18% LiCl content. Consequently, the substantial difference observed in the specific surface area suggested that changes occurred within the internal structures of the microspheres. These findings indicated a considerable effect of LiCl incorporation on the inner structure of the composite microspheres.

Building on established research^25^, this study confirmed that the Mg–Al layered hydroxide materials, synthesized via co-precipitation, consisted of nanoparticle flakes. This morphology resulted in the formation of numerous internal voids during the subsequent spray drying stages. The data presented in Fig. 3d indicate a trend of both the average pore size and porosity of the composite microspheres gradually decreasing with increasing LiCl content. This trend likely originated from the precipitation of lithium salts during the drying phase, which occupied the voids within the microspheres^26^. Specifically, at a lower LiCl threshold (≤ 10%), the microspheres maintained high porosity levels (> 0.39 cm^3^/g) with pore sizes exceeding 35 nm. Conversely, at an LiCl concentration of 26%, an excess of lithium salt resulted in nearly closed internal pores, thus reducing the porosity to 0.016 cm^3^/g and decreasing the pore diameter to approximately 11.98 nm.

**Figure 4:**
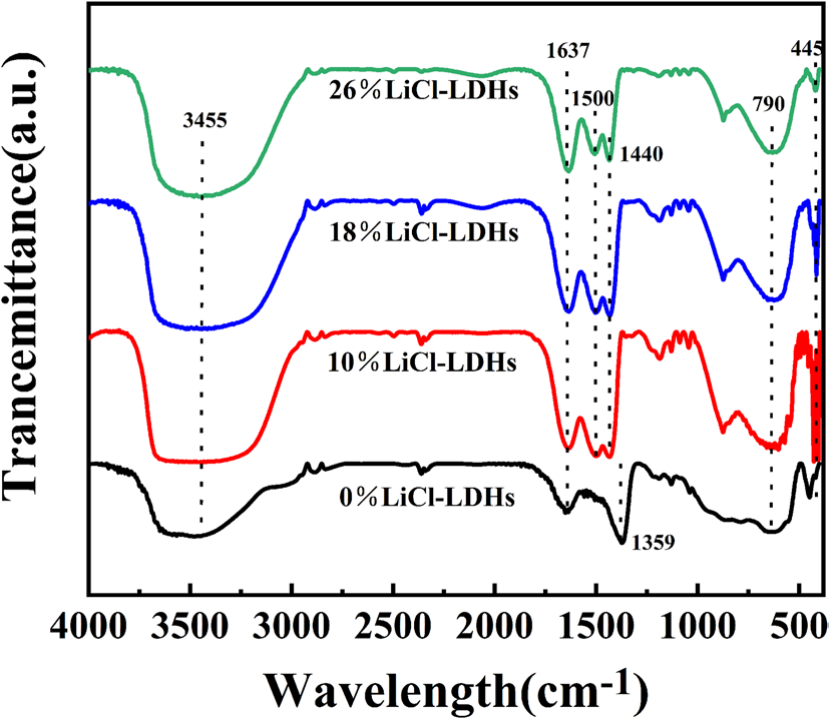
FT-IR curves of LiCl-LDHs composite microspheres with different LiCl content.

The FT-IR spectra of the LiCl-LDHs composite microspheres, recorded over a 400–4000 cm^−1^ range, are illustrated in Fig. 4. The peak approximately at 3455 cm^−1^ was attributed to the O–H stretching vibrations, suggesting the presence of surface-adsorbed and interlayer water within the samples^27^. The broadening phenomenon of this peak, correlated with an increase in LiCl content, indicated an enhanced water retention capacity that was attributable to the hygroscopic nature of LiCl. A peak emerged at 1637 cm^−1^ due to the bending vibrations of crystalline water^28^. The band at 1500 cm^−1^ possibly resulted from interactions between OH^−^ ions and Li^+^^29^. Moreover, the distinct peak proximate to 1359 cm^−1^ could be attributed to the C–O stretching vibrations, which indicated the intercalation of CO_3_^2−^ in the LDH layers, consisting of a byproduct of atmospheric CO_2_ exposure during sample handling^30^. Upon addition of LiCl, the vibrational peak at 1359 cm^−1^ disappears and transforms into a small shoulder at 1400 cm^−1^, indicating a decrease in the symmetry of the carbonate anion. This is most likely due to some interaction between the free carbonate anion and its surroundings^31^. The spectral region ranging from 500 to 790 cm^−1^ included the translational and deformation modes of Al–OH bonds, and the band observed near 445 cm^−1^ corresponded to the O–M–O (where M = Mg or Al) lattice vibrations within the octahedral layers of Mg–Al LDHs^32^. The maintenance of these signature infrared features across the spectrum confirmed that the core chemical structure of the Mg–Al LDHs material remained intact despite the incorporation of LiCl.

**Figure 5:**
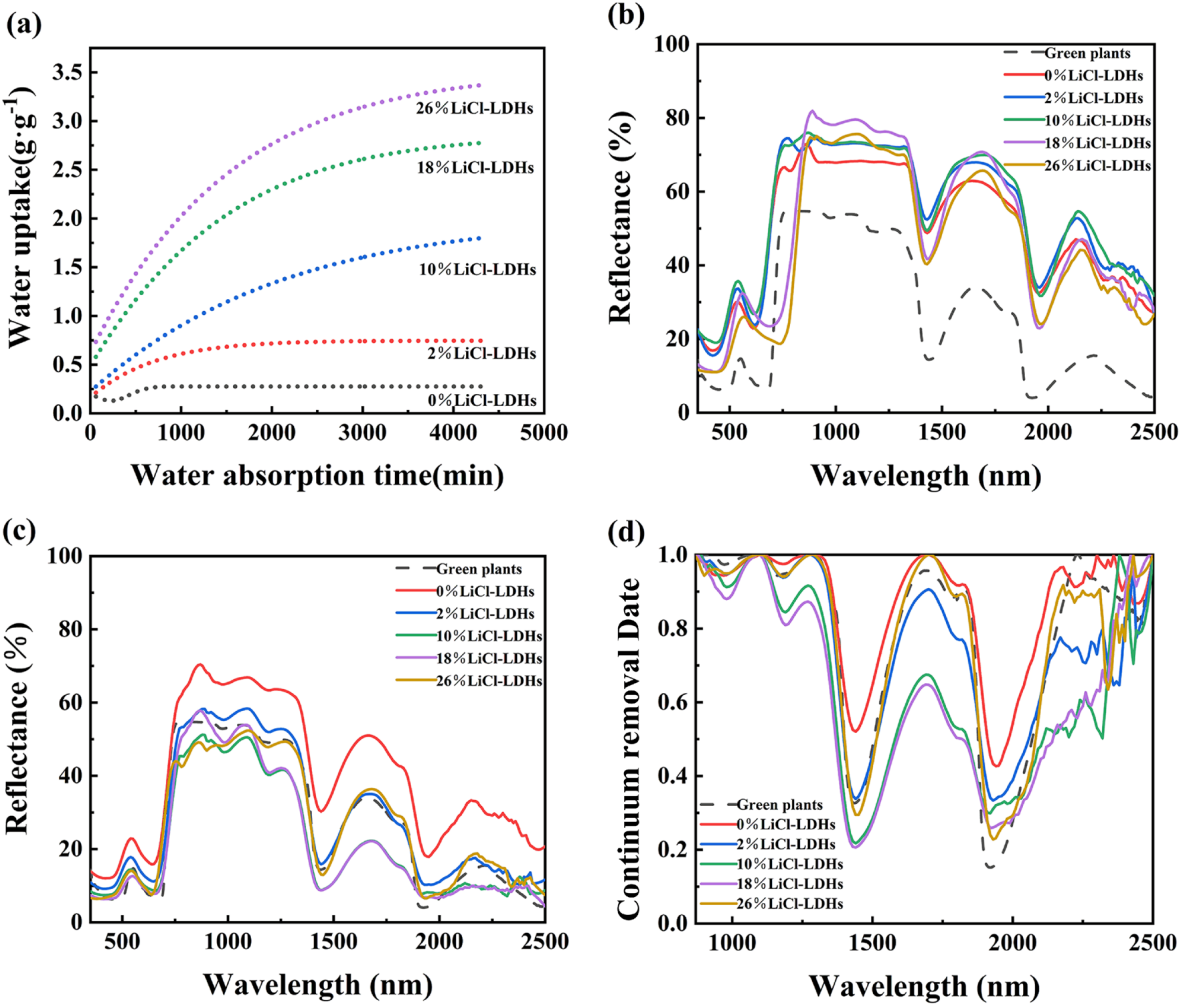
Water absorption ratio curves and spectral curves of LiCl-LDHs composite microsphere with different LiCl content [(**a**) hygroscopic curves; (**b**) spectral curves of dry powders; (**c**) spectral curves after saturation moisture absorption; (**d**) continuum removal spectrum after saturation moisture absorption].

To clarify the influence of LiCl content on the moisture uptake efficacy of Mg–Al LDHs, the hygroscopic profiles of different LiCl-LDHs composite microspheres were assessed under conditions of constant temperature and elevated humidity. As shown in Fig. 5a, a direct correlation was evident between the LiCl proportion in the composite and its corresponding water-loading capacity. Significantly, the sample without LiCl exhibited the lowest saturation moisture uptake ratio, registering only 0.32 g/g. The homogeneous distribution of LiCl, even in modest amounts, in the microsphere pores notably enhanced the moisture absorption capacity of the composite material, thus simultaneously improving the water-loading capacity of the internal micro-pores and interfaces. During the initial hygroscopic stage, adsorbed water was present in the form of solid crystalline hydrates. With increasing water content, a saline solution progressively formed and permeated the internal pores of the microspheres, endowing the material with the ability to chemically absorb moisture^33^. According to this hygroscopic mechanism, the greater the LiCl content in the composite microsphere, the more pronounced its hygroscopic ability. However, with the inclusion of 26% LiCl, the maximum saturation moisture uptake ratio reached 3.4 g/g. Nevertheless, the absorbed water was not limited to the internal pores of the microspheres but also formed a thin film on the surface of the particles, indicating a deliquescence phenomenon, which led to powder agglomeration.

To examine the effect of LiCl content on the optical properties of the composite microspheres in greater detail, the visible and near-infrared reflectance spectra of the LiCl-LDHs composite microspheres before and after moisture absorption were analyzed. Figure 5b shows the visible and near-infrared spectral curves of the LiCl-LDHs samples in a desiccated state. Although the pure Mg–Al LDHs microspheres displayed spectral characteristics analogous to green leaves, the overall spectral reflectance was substantially higher than that of natural foliage. Moreover, the water absorption peak intensity of the desiccated microspheres was noticeably subdued, indicating significantly lower water content relative to natural plants. When a minimal quantity of LiCl (≤ 10%) was introduced to the microspheres, the spectral shape of the desiccated samples was largely retained. However, due to the high reflectance of LiCl, the spectral reflectance of the composite microspheres experienced a minor elevation. With increasing LiCl concentrations (≥ 18%), the position of the red edge was found to gradually shift toward longer wavelengths, spanning from 630 to 750 nm. This shift likely resulted from the precipitation of LiCl particles onto the surfaces of the microspheres with elevated LiCl content, resulting in a light scattering effect that intensified light absorption in the 700–760 nm range. Furthermore, an enhanced water peak intensity in the near-infrared band was indicative of the tendency of the microspheres to absorb moisture from the surrounding air.

Figure 5c presents the spectral curves of the LiCl-LDHs composite microspheres following complete moisture absorption. Each sample was saturated with moisture after 74 h. For LDHs samples without LiCl, the spectral shape and reflectance exhibited negligible variations due to weak hygroscopicity. By contrast, incorporating LiCl into the LDHs materials resulted in increased water content, which altered the material's refractive index and markedly reduced the spectral reflectance across the entire visible and near-infrared band. Using the sample with 2% LiCl content as an example, the average spectral reflectance at 400–2500 nm was found to decrease from 66 to 58% after hygroscopic absorption. With increasing LiCl content, the water content in the sample also increased, leading to a further reduction in spectral reflectance. Notably, the red edge position of the sample experienced only a 20 nm redshift following hygroscopic absorption, and the spectral shape became more akin to that of a green leaf. Specifically, the near-infrared reflectance of the sample was slightly lower than that of natural plants, which was attributed to the increased water content in the sample after hygroscopic absorption. The absorbed water dissolved some of LiCl, diminishing its influence on spectral characteristics. However, water also modified the refractive index of the composite particles to a degree, thus enhancing the scattering and absorption of incident light. Ultimately, the visible light spectra of the composite microsphere samples closely matched that of plant leaves, while the reflectance of the near-infrared spectrum remained noticeably lower than that of plant leaves.

To facilitate the visual comparison of the intensity variations among characteristic absorption peaks, the reflectance spectral curve of the sample was subjected to envelope removal. As shown in Fig. 5d, at lower LiCl concentrations, the intensity of the water absorption peaks near 1450 and 1940 nm correlated with the concentration of LiCl. The water peak intensities for the 10% and 18% LiCl-LDHs composite microspheres were found to be relatively high. However, with further increases in LiCl content, the intensities of the water absorption peaks diminished. This phenomenon could be attributed to excess lithium salt, which caused a significant amount of brine solution to form not only within the microporous material but also on the surface of the powder^34^. However, to prevent instrument contamination during spectral testing, the removal of water on the coating surface was imperative. As a result, the water content indicated by the spectral test results was substantially lower than the actual water content in the sample. Along with the analysis of the microsphere morphology and structure, the comprehensive performance of the 10% LiCl-LDHs samples proved to be superior, demonstrating the closest resemblance to the visible and near-infrared spectra of natural plants. At this point, the spectral similarity index, *r*_*xy*_, achieved a value of 95.58%.

**Figure 6:**
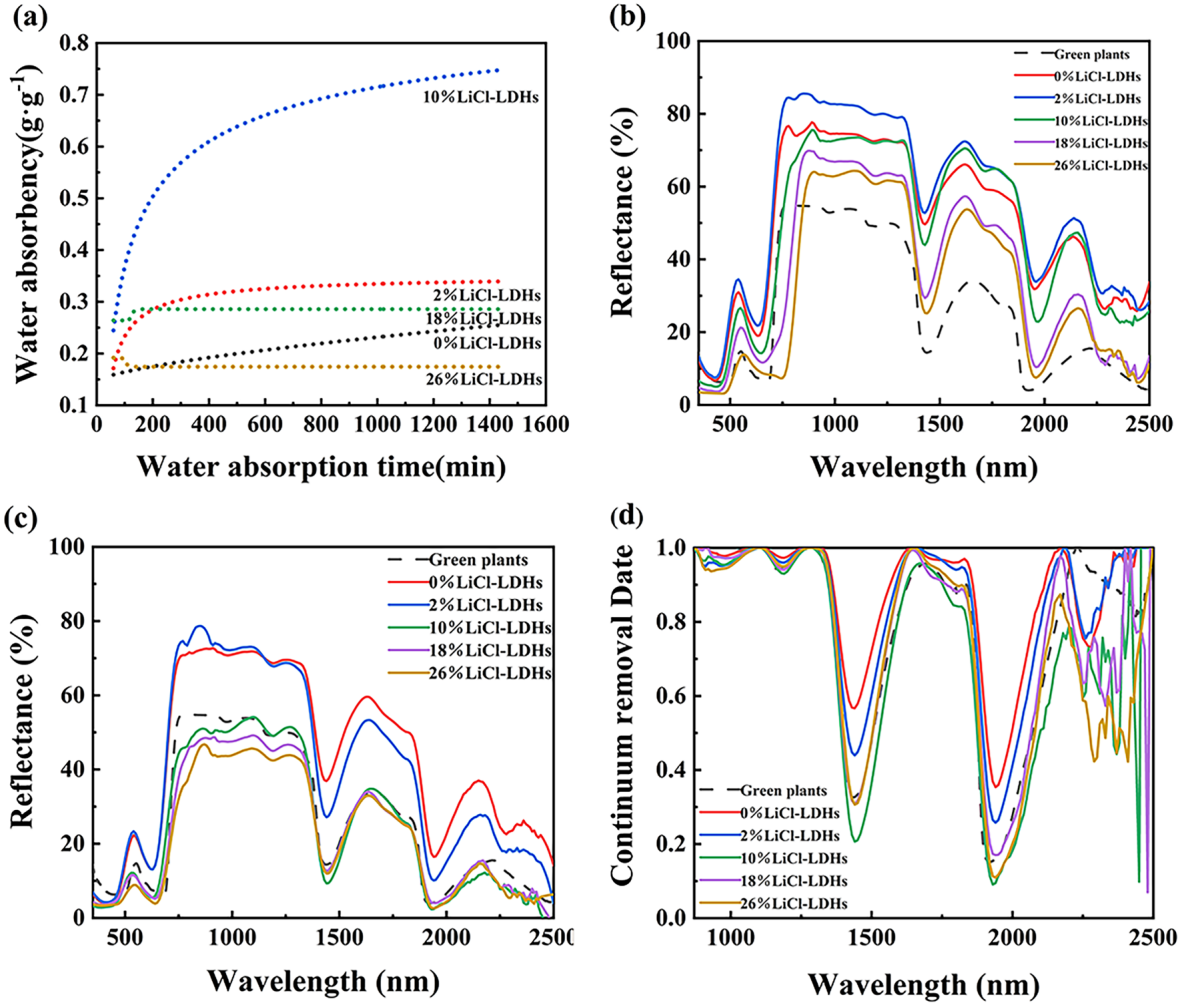
Water absorption ratio curves and spectral curves of composite coating with different LiCl content [(**a**) hygroscopic curves; (**b**) spectral curves of dry coating; (**c**) spectral curves after saturation moisture absorption; (**d**) continuum removal spectrum after saturation moisture absorption].

Subsequently, coating samples were fabricated utilizing x% LiCl-LDHs microsphere pigments, and their hygroscopic curves and reflectance spectra were evaluated. As illustrated in Fig. 6a, the coating fabricated with a 2% LiCl-LDHs pigment exhibited a saturation moisture uptake ratio of 0.34 g/g. Compared with the coating without LiCl (0.26 g/g), the moisture uptake was marginally enhanced. The coating sample incorporating 10% LiCl-LDHs pigment demonstrated the highest moisture uptake capability, with a maximum saturation moisture uptake ratio of 0.75 g/g. With further increases in LiCl content, the moisture uptake capacity of the coating diminished. The saturation moisture uptake ratio for the coating with 26% LiCl-LDHs was only 0.18 g/g. The water absorption ratio of the coating was noticeably lower than that of the LiCl-LDHs powder because the composite microsphere particles were uniformly embedded in the resin, and their surfaces were entirely encased by acrylic resin. The microsphere particles could only interact with atmospheric moisture via confined pathways, thus significantly constraining the hygroscopic process.

**Figure 7:**
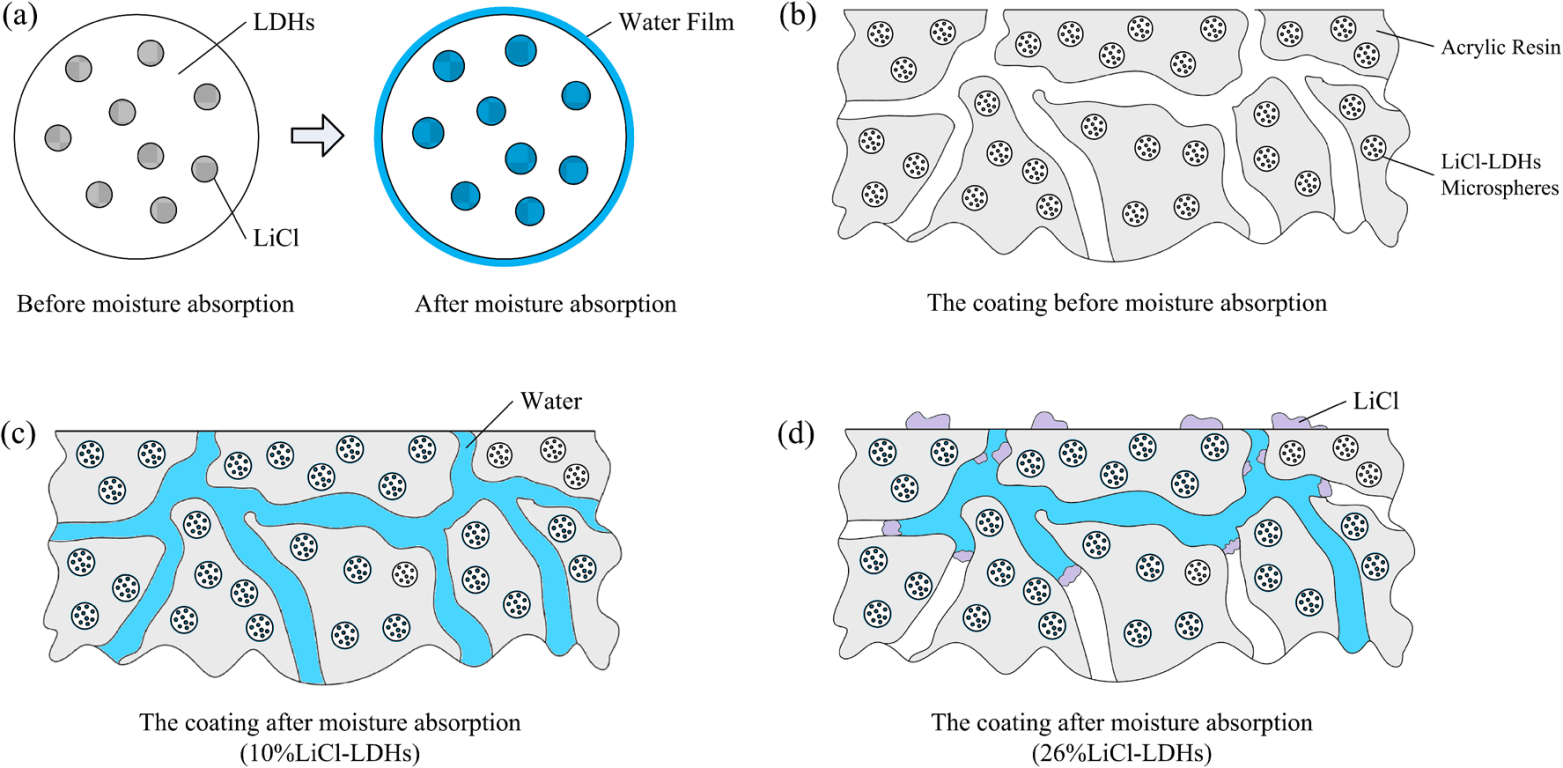
Schematic diagram of hygroscopic mechanism (**a**) LiCl-LDHs composite microspheres; (**b**) the coating before moisture absorption; (**c**,**d**) the coating after moisture absorption).

When the coating encompassed an optimal amount of LiCl, water could permeate the coating through the pores and could be subsequently absorbed by the composite microspheres and retained within their structure. However, when the LiCl concentration exceeded a certain threshold, an excessive quantity of lithium salt became intermixed with the LDHs material and could also coat the surface of the microspheres. Upon water absorption, the lithium salt progressively dissolved, and excess salt solution could not be sustained at the powder-resin interface, necessitating its migration toward the coating surface through the pores. Throughout this process, the significant depletion of lithium salt diminished the effective LiCl concentration in the coating, consequently leading to a reduction in its water absorption capacity. In addition, the migration of LiCl may also block the internal channels of atmospheric moisture into the coating, thereby reducing the water absorption capacity of composite particles and pores. The relevant influence mechanisms are shown in Fig. 7.

As indicated by the spectral curve in Fig. 6, the influence of LiCl on the spectral properties of the dry coating was the same as that the composite particles. With an increase in LiCl content, a gradual reduction in spectral reflectance was observed, accompanied by a pronounced redshift in the spectral characteristics of the red edge. All the coated samples were subjected to moisture absorption for 24 h and all the samples were saturated with moisture absorption rate. After subjecting the coating material to saturated moisture absorption, the spectral reflectance diminished further, aligning with the change in spectral curve with the water absorption characteristics. As illustrated in Fig. 6d, when the LiCl concentration in the coating was 10%, the peak of spectral water absorption was most pronounced, and the spectral profile more closely resembled that of natural vegetation, with a spectral curve similarity reaching an impressive value of 97.41%.

**Figure 8:**
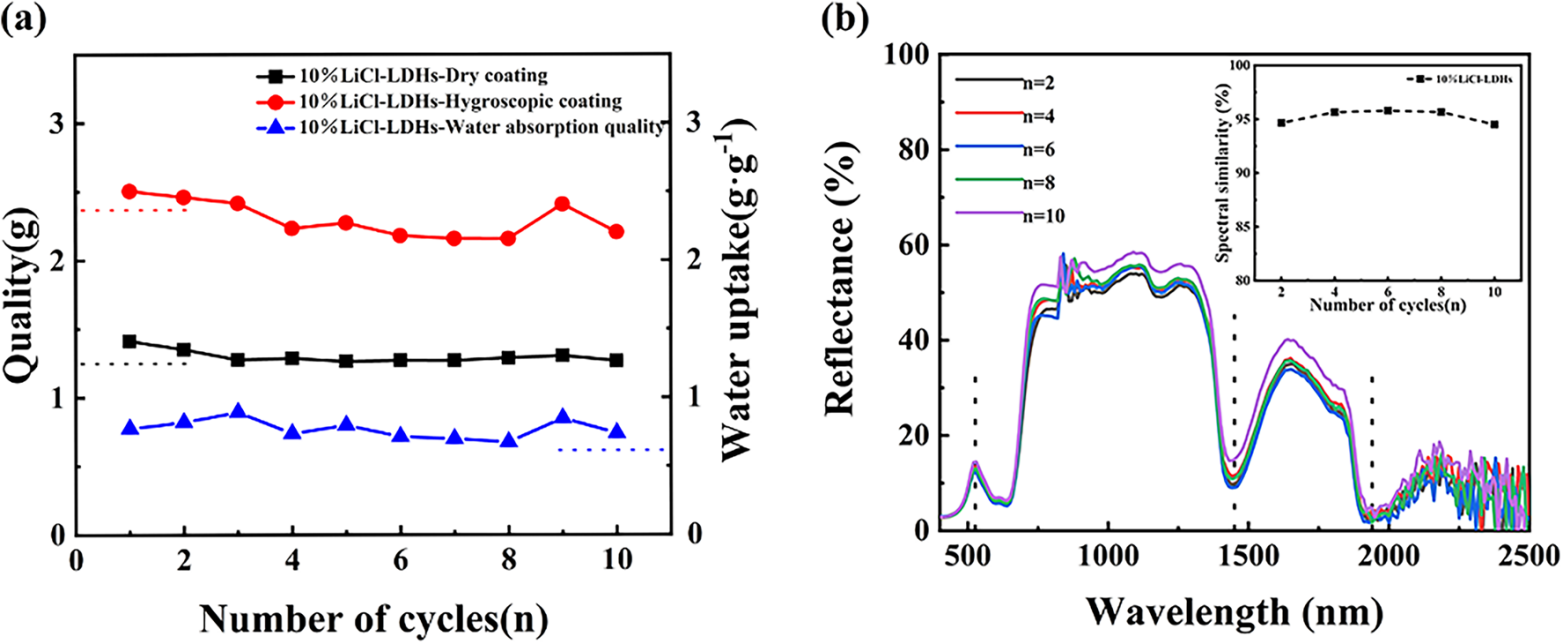
Mass change data (**a**) and reflection spectral curves (**b**) of 10% LiCl-LDHs composite coating after *n* times of drying and moisture absorption cycle experiment.

To determine the cyclic hygroscopicity of the composite coating, a series of 10 drying and moisture absorption cycles was performed on the 10% LiCl-LDHs coating samples. Figure 8a presents the mass fluctuation curve of the sample following repeated drying and hygroscopic cycle treatment. Despite observed fluctuations in the mass of the coating, it retained a relatively consistent moisture absorption capacity, and the water absorption ratio was generally sustained at approximately 0.77 g/g. As indicated by the spectral curve in Fig. 8b, repeated drying and moisture absorption treatment did not significantly change the visible–near-infrared reflectance spectrum of the coating. The spectral congruence between the samples and natural vegetation remained at approximately 95%. This suggested that the composite material exhibited robust resilience to drastic changes in ambient temperature and humidity, demonstrating considerable environmental adaptability.

**Figure 9:**
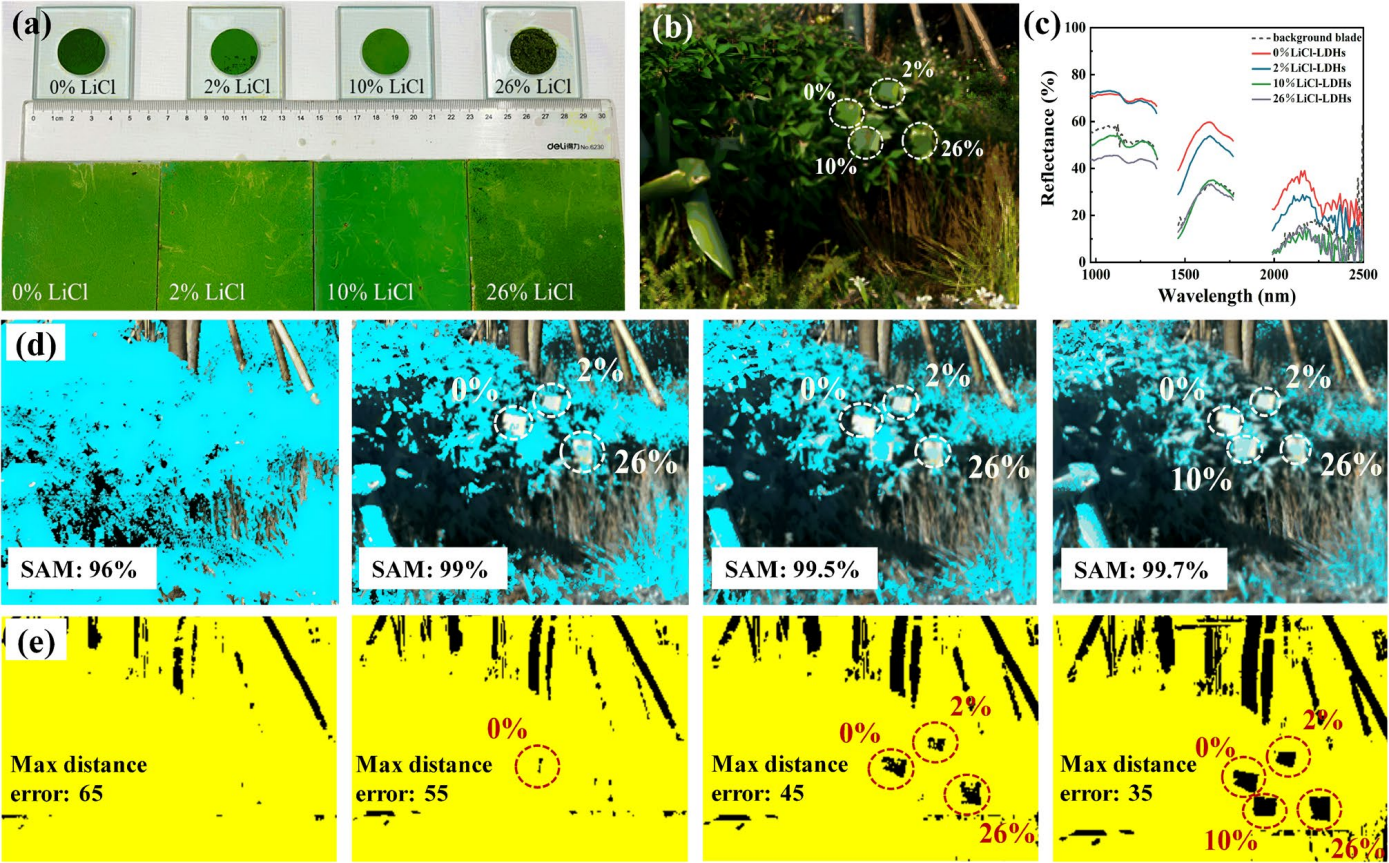
Concealment of LiCl-LDHs coatings in the hyperspectral image [(**a**) the optical images of LiCl-LDHs powders and coatings; (**b**) the optical images of LiCl-LDHs coatings in the leaf background; (**c**) NIR spectral curves extracted from the hyperspectral image; (**d**) SAM classification results of camouflage coatings in leaf background with different thresholds; (**e**) MD classification results of camouflage coatings in leaf background with different threshold].

Finally, the camouflage efficacy of the coatings in the context of hyperspectral imaging was illustrated, as shown in Fig. 9. Attempts were made to detect the coatings positioned against a foliar background employing a hyperspectral camera. Figure 9c shows the near-infrared spectral curves (900–2500 nm) derived from the hyperspectral image. Analysis revealed that the near-infrared spectrum of the 10% LiCl-LDH coatings demonstrated the closest resemblance to that of natural vegetation in terms of both spectral shape and intensity.

To advance the verification of the spectral discrepancy effects on near-infrared hyperspectral camouflage, spectral angle mapping (SAM) and Mahalanobis distance (MD) mapping classifications were employed to assess the similarity between the coatings and the plant backdrop. SAM constituted a physically grounded spectral classification method that used an n-dimensional angle for aligning pixels with the reference spectra^35^. By setting a cosine similarity threshold, precise mapping of the areas with a higher cosine similarity to the reference spectra could be achieved. This technique focused on discerning the spectral shape disparities between the target and the backdrop within hyperspectral images. In contrast to SAM, MD was characterized by calculating the MD to quantify the disparity between the sample spectrum and background reference spectrum^36^. Through the application of a defined maximum distance threshold, targets with spectral distances exceeding this margin could be distinguished in the hyperspectral images, which primarily captured variances in reflectance between the target and background spectra.

As shown in Fig. 9d, setting the threshold at 96.0% resulted in an accurate classification of most genuine leaves, where all four samples were concurrently mistaken for foliage. An increase in the threshold value decreased the extent of the mask, resulting in a more exact classification. At a threshold of 99%, the 0% LiCl-LDHs, 2% LiCl-LDHs, and 26% LiCl-LDHs samples were effectively differentiated from the mask, thus standing out against the background. Complete isolation of the 10% LiCl-LDHs sample required the threshold to be raised to 99.7%. However, this high threshold introduced an overfitting issue, where a significant number of green leaves were misclassified as false, alongside the 10% LiCl-LDHs sample, thereby rendering this criterion untenable for distinguishing between the camouflage coating and real leaves.

The classification results obtained via the MD are illustrated in Fig. 9e. When the maximum distance error threshold was set to 65, all samples remained indistinguishable against the background. Due to the most pronounced spectral reflectance difference between the 0% LiCl-LDHs sample and natural leaves, the former was partially detectable at a maximum distance error of 55, while the latter three samples continued to be classified as plant leaves. After reducing the maximum distance error to 45, the 2% LiCl-LDHs and 26% LiCl-LDHs samples became distinguishable from the background, while the 10%LiCl-LDHs sample continued to blend with the background. The 10% LiCl-LDHs sample remained undetected against the background until the threshold was further lowered to 35.

In summary, the superior hyperspectral camouflage capabilities of the 10% LiCl-LDHs coating were corroborated in practical scenarios. Unlike traditional optical camouflage materials, which may be readily detectable, an optimal concentration of LiCl enhanced the water content of the coating, which contributed to the augmented scattering and absorption effects of near-infrared light. The intensity and shape of the spectrum more closely resembled those of natural leaves, indicating significant potential for application in hyperspectral camouflage.

## Conclusion

Artificial camouflage materials inspired by natural camouflage mechanisms have evolved in response to the demands of military technology. Recent advancements in spectral imaging technology for target detection have driven the evolution of camouflage technology toward greater dimensionality and precision. To effectively conceal military targets against a background, the VIS and NIR spectral properties of camouflage materials must be precisely tailored to mimic their surroundings with high fidelity.

In this work, the LiCl-LDHs composite microspheres were synthesized using a spray granulation technique and served as a functional pigment in the hyperspectral camouflage coatings, achieving the accurate simulation and control over the visible to near-infrared spectral trait characteristics of green foliage. After the spray granulation process, the LiCl-LDHs powder displayed spherical particles with smooth surfaces, and LiCl was primarily localized in the internal cavities of the Mg–Al LDHs microspheres. LiCl significantly enhanced the hygroscopic properties of the composite microspheres, which were positively correlated with the LiCl content. However, an excessive concentration of LiCl could extend the spray drying process, potentially compromising the crystalline structure and particle morphology of the Mg–Al LDHs microspheres. The results indicated that the camouflage coating containing 10% LiCl-LDHs composite microspheres demonstrated the highest water absorption capacity. When compared with coatings lacking LiCl, the water absorption ratio could be increased from 0.26 to 0.75 g/g. This composite microsphere material featured the individual tunability of red edge and NIR reflectance, which mimicked the spectral variation of natural leaves in the realistic canopy. The spectral match between the coating and natural foliage could reach 97.41%, suggesting significant potential for hyperspectral camouflage applications across the VIS to NIR spectra. Additionally, the coating demonstrated good environmental adaptability, preserving its VIS and NIR spectral properties even after undergoing 10 cycles of drying and moisture absorption testing.

Ultimately, the LiCl-LDHs composite microspheres endowed the artificial coating with robust camouflage capabilities against hyperspectral target detection, which could be mistaken for foliage by the SAM and MD mappers to an overfitting level. This novel material design approach may provide a new method for the development of high-performance hyperspectral camouflage materials.

## Data Availability

The datasets generated and/or analysed during the current study are not publicly available due but are available from the corresponding author on reasonable request.

## References

[1] Lv C, Zu M, Xie D, Cheng H (2021). Emulating solar spectral reflectance of natural leaf with bionic leaf prepared from 4A zeolite-derived ultramarine green pigment. Materials.

[2] Gates DM, Keegan HJ, Schleter JC, Weidner VR (1965). Spectral properties of plants. Appl. Opt..

[3] Sims DA, Gamon JA (2003). Estimation of vegetation water content and photosynthetic tissue area from spectral reflectance: A comparison of indices based on liquid water and chlorophyll absorption features. Remote Sens. Environ..

[4] Liu Z, Wu W, Hu B (2008). Design of biomimetic camouflage materials based on angiosperm leaf organs. Sci. China Ser. E Technol. Sci..

[5] Yang Y, Hu B, Wu W (2011). Design and preparation of bionic camouflage materials by simulating plant leaves. J. Natl. Univ. Def. Technol. NUDT.

[6] Lv X, Yuan L, Rao C, Wu X, Qing X, Weng X (2023). Structure and near-infrared spectral properties of mesoporous silica for hyperspectral camouflage materials. Infrared Phys. Technol..

[7] Evans D, Xue D (2006). Preparation of layered double hydroxides and their applications as additives in polymers, as precursors to magnetic materials and in biology and medicine. Chem. Commun..

[8] Zhao J, Chen J, Xu S, Shao M, Yan D, Wei M, Evans DG, Duan X (2013). CoMn-layered double hydroxide nanowalls supported on carbon fibers for high-performance flexible energy storage devices. J. Mater. Chem. A.

[9] Li S, Ribeiro AM, Shi Y, Moreira MN, Cai N, Rodrigues AE (2015). Synthesis, pelleting, and performance evaluation of a novel K-promoted γ-alumina/MgAl-layered double oxide composite adsorbent for warm gas H_2_/CO_2_ separation. Ind. Eng. Chem. Res..

[10] Wang G, Rao D, Li K, Lin Y (2014). UV blocking by Mg–Zn–Al layered double hydroxides for the protection of asphalt road surfaces. Ind. Eng. Chem. Res..

[11] Zhu H, Tang P, Feng Y, Wang L, Li D (2012). Intercalation of IR absorber into layered double hydroxides: Preparation, thermal stability and selective IR absorption. Mater. Res. Bull..

[12] Buffet JC, Wanna N, Arnold TAQ, Gibson EK, Wells PP, Wang Q, Tantirungrotechai J, O’Hare D (2015). Highly tunable catalyst supports for single-site ethylene polymerization. Chem. Mater..

[13] Kamyar A, Khakbiz M, Zamanian A, Yasaei M, Yarmand B (2019). Synthesis of a novel dexamethasone intercalated layered double hydroxide nanohybrids and their deposition on anodized titanium nanotubes for drug delivery purposes. J. Solid State Chem..

[14] Miao S, Luo Z, Tan S, Xu T, Zhou Z, Feng G, Xu G, Ji G (2024). Mg–Al layered double hydroxides film coating for efficient biomimetic stealth. Prog. Org. Coat..

[15] Xu J, Li T, Chao J, Wu S, Yan T, Li W, Cao B, Wang R (2020). Efficient solar-driven water harvesting from arid air with metal–organic frameworks modified by hygroscopic salt. Angew. Chem..

[16] Liu JY, Wang JY, Wang LW, Wang RZ (2017). Experimental investigation on properties of composite sorbents for three-phase sorption-water working pairs. Int. J. Refrig..

[17] Sun Y, Spieß A, Jansen C, Nuhnen A, Gökpinar S, Wiedey R, Ernst S-J, Janiak C (2020). Tunable LiCl@UiO-66 composites for water sorption-based heat transformation applications. J. Mater. Chem. A.

[18] Hou Y, Sheng Z, Fu C, Kong J, Zhang X (2022). Hygroscopic holey graphene aerogel fibers enable highly efficient moisture capture, heat allocation and microwave absorption. Nat. Commun..

[19] Qin R, Xu G, Guo L, Jiang Y, Ding R (2012). Preparation and characterization of a novel poly(urea–formaldehyde) microcapsules with similar reflectance spectrum to leaves in the UV–Vis–NIR region of 300–2500 nm. Mater. Chem. Phys..

[20] Cantrell DG, Gillie LJ, Lee AF, Wilson K (2005). Structure-reactivity correlations in MgAl hydrotalcite catalysts for biodiesel synthesis. Appl. Catal. A Gen..

[21] Chen C, Zhang Z, Liu J, Li Q, Zhang Y, Xiong J (2019). Regulating the dissociation of LiCl and transportation of Li ions within UiO-66-NH_2_ framework for humidity sensing applications with superb comprehensive performances. J. Alloys Compd..

[22] Chen YR, Liou KH, Kang DY, Chen JJ, Lin LC (2018). Investigation of the water adsorption properties and structural stability of MIL-100(Fe) with different anions. Langmuir.

[23] Shkatulov A, Gordeeva LG, Girnik IS, Huinink H, Aristov YI (2020). Novel adsorption method for moisture and heat recuperation in ventilation: Composites “LiCl/matrix” tailored for cold climate. Energy.

[24] Tan B, Luo Y, Liang X, Wang S, Fang Y (2019). Composite salt in MIL-101(Cr) with high water uptake and fast adsorption kinetics for adsorption heat pumps. Microporous Mesoporous Mater..

[25] Yuan L, Wang C, Qing X, Bi M, Huang G, Weng X (2020). Synthesis and fine spectroscopy tuning of the hyperspectral simulation material based on organic anions intercalated Mg–Al layered double hydroxide. Infrared Phys. Technol..

[26] Zhao H, Lei M, Liu T, Huang T, Zhang M (2020). Synthesis of composite material HKUST-1/LiCl with high water uptake for water extraction from atmospheric air. Inorg. Chim. Acta.

[27] Zhang F, Zhang C, Song L, Zeng R, Liu Z, Cui H (2015). Corrosion of in-situ grown MgAl-LDH coating on aluminum alloy. Trans. Nonferr. Met. Soc. China.

[28] Rahman MT, Kameda T, Miura T, Kumagai S, Yoshioka T (2020). Facile method for treating Zn, Cd, and Pb in mining wastewater by the formation of Mg–Al layered double hydroxide. Int. J. Environ. Sci. Technol..

[29] Serizawa N, Takei K, Nishikiori T, Katayama Y, Ito Y (2018). Infrared spectra of N–H compounds in LiCl-KCl-CsCl molten salts using the diffuse reflectance optical system. Electrochemistry.

[30] Li C, Wu S, Chen Z, Shu B, Li Y, Xiao Y, Liu Q (2019). Synthesis of Fe_3_O_4_-decorated Mg–Al layered double hydroxides magnetic nanosheets to improve anti-ultraviolet aging and microwave absorption properties used in asphalt materials. Constr. Build. Mater..

[31] Faramawy S, Zaki T, Sakr AA-E, Saber O, Aboul-Gheit AK, Hassan SA (2018). The activity of Mg–Al layered double hydroxides intercalated with nitrogen-containing anions towards the removal of carbon dioxide from natural gas. J. Nat. Gas Sci. Eng..

[32] Wu L, Pan F, Liu Y, Zhang G, Tang A, Atrens A (2018). Influence of pH on the growth behaviour of Mg–Al LDH films. Surf. Eng..

[33] Zhao H, Wang Z, Li Q, Wu T, Zhang M, Shi Q (2020). Water sorption on composite material “zeolite 13× modified by LiCl and CaCl_2_”. Microporous Mesoporous Mater..

[34] Yang B, Wang C, Ji X, Yue X, Lv G, Wang M (2022). Investigation on water vapor adsorption performance of carbon based composite adsorption material ACF-silica sol-LiCl. Microporous Mesoporous Mater..

[35] Weyermann J, Schläpfer D, Hueni A, Kneubuehler M, Schaepman M (2009). Spectral angle mapper (SAM) for anisotropy class indexing. Imaging Spectrom..

[36] Panda A, Pachori RB, Sinnappah-Kang ND (2021). Classification of chronic myeloid leukemia neutrophils by hyperspectral imaging using Euclidean and Mahalanobis distances. Biomed. Signal Process. Control.

